# Mobile health for mental health: a pilot study on well-being, burnout, and stress among healthcare professionals

**DOI:** 10.3389/fpsyg.2026.1867560

**Published:** 2026-07-01

**Authors:** Simona Dobešová Cakirpaloglu, Panajotis Cakirpaloglu, Barbora Kvapilová, Tereza Schovánková, Ondrej Skopal, Śárka Vévodová

**Affiliations:** 1 Department of Humanities and Social Sciences, Faculty of Health Science, Palacký University Olomouc, Olomouc, Czechia; 2 Department of Psychology, Faculty of Arts, Palacký University Olomouc, Olomouc, Czechia; 3 Department of Psychology and Abnormal Psychology, Faculty of Education, Palacký University Olomouc, Olomouc, Czechia; 4 Doctoral Studies Office, Faculty of Health Sciences, Palacký University Olomouc, Olomouc, Czechia

**Keywords:** burnout, CBT, digital intervention, healthcare professionals, mental well-being, mHealth, mindfulness, stress

## Abstract

**Background:**

Mobile health (mHealth) applications have the potential to improve mental well-being, reduce burnout, and manage stress among healthcare professionals. However, evidence on their effectiveness remains limited.

**Objective:**

This pilot study evaluated preliminary changes in mental well-being, perceived stress, and burnout associated with the use of *CalmAnna* among healthcare professionals.

**Methods:**

Using a single-group pre–post design, this study was conducted with 75 healthcare professionals who used the *CalmAnna* mobile application over a six-week period. The application includes evidence-based modules – such as mindfulness, relaxation techniques, and cognitive-behavioral therapy (CBT)-inspired exercises, supplemented by psychoeducational content. Study outcomes were assessed at baseline and at six weeks using validated self-report measures (WHO-5, MBI, PSS-10, SUPSO-7), administered both online and via paper-and-pencil formats. In addition, participants completed an in-app self-assessment of stress, burnout, and well-being, which was used to generate a personalized intervention plan.

**Results:**

A total of 75 healthcare professionals were included in the analysis. Significant improvements were observed in mental well-being (Δ = +1.55, 95% CI [0.61, 2.48], *p* = 0.001) and reductions in perceived stress (Δ = −1.73, 95% CI [−2.40, −1.06], *p* < 0.001). Burnout indicators also showed significant improvements, including reductions in emotional exhaustion and depersonalization and an increase in personal accomplishment. Changes across psychological state dimensions (SUPSO) indicated increased positive and decreased negative states. All reported effects remained significant after correction for multiple comparisons.

**Conclusions:**

*CalmAnna* appears to be a promising mHealth intervention associated with improvements in mental well-being and reductions in stress and burnout among healthcare professionals. Further randomized controlled studies with larger samples and longer follow-up periods are needed to establish its effectiveness.

## 1 Introduction

Healthcare professionals, including physicians and nurses, experience some of the highest levels of chronic stress, emotional exhaustion, and burnout worldwide (Tennant et al., 2007; Maslach et al., 1996). Recent evidence further indicates that burnout affects a substantial proportion of healthcare workers, with prevalence rates frequently exceeding 30%−50% and even higher levels reported in specific specialties (e.g., Dobešová Cakirpaloglu et al., 2026; Dudina and Martinsone, 2025). Burnout is conceptualized as a psychological syndrome resulting from prolonged exposure to workplace stressors, characterized by emotional exhaustion, depersonalization, and reduced professional accomplishment (Maslach et al., 1996; Dobešová Cakirpaloglu et al., 2026). They are exposed to a wide range of occupational stressors, such as high workload, shift work, fast-paced environments, moral conflicts, and emotionally demanding patient care (Søvold et al., 2021). Recent evidence further highlights high workload, insufficient organizational support, interpersonal conflict, and poor work–life balance as key contributors to burnout among healthcare workers (Søvold et al., 2021; West et al., 2018; Shanafelt et al., 2022). These factors, often combined with insufficient organizational support and workplace challenges such as limited psychological safety or interpersonal conflicts, contribute to increased psychological strain and reduced well-being among healthcare workers. Prolonged exposure to such stressors may lead to adverse mental health outcomes, including depression, anxiety disorders, sleep disturbances, and burnout (Dobešová Cakirpaloglu et al., 2024).

The consequences of these conditions extend beyond individual health and significantly affect healthcare systems. Reduced well-being among healthcare professionals has been associated with decreased quality of care, lower patient safety, increased absenteeism, and higher staff turnover (Chen and Meier, 2021; Kumar, 2016). In recent years, global crises, including the COVID-19 pandemic and ongoing geopolitical instability, have further intensified these challenges, highlighting the urgent need to support the mental health of healthcare workers (Safiye et al., 2023). Empirical evidence consistently demonstrates that psychological distress in healthcare professionals is linked to impaired work performance, reduced productivity, and difficulties in fulfilling daily professional responsibilities (Hussein et al., 2024).

Chronic exposure to high job demands may lead to the development of burnout, absenteeism, and presenteeism, all of which negatively affect job performance and organizational outcomes (Silva-Costa et al., 2020). Burnout has been defined in the International Classification of Diseases (ICD-11) as a syndrome resulting from chronic workplace stress that has not been successfully managed, characterized by emotional exhaustion, increased mental distance from one's job, and reduced professional efficacy (World Health Organization, 2019). In addition, presenteeism—defined as working despite physical or psychological distress—has been associated with psychosocial risk factors such as high job demands, low reward, limited job control, and insufficient social support (Silva-Costa et al., 2020). These conditions increase the risk of developing mental health problems, including depression and anxiety, and negatively affect both personal and professional functioning. Previous research has also demonstrated a strong relationship between burnout and depression, particularly in relation to emotional exhaustion (Dobešová Cakirpaloglu et al., 2024).

The increasing prevalence of burnout among healthcare professionals has been documented globally, particularly among physicians exposed to long-term and severe stressors (Kumar, 2016). These individuals often report high levels of emotional exhaustion, depersonalization, compassion fatigue, and reduced personal accomplishment. Such outcomes may contribute to a range of negative consequences, including mental health disorders, sleep disturbances, substance use, and impaired social relationships, in some cases leading to suicidal ideation (Chen and Meier, 2021; Kumar, 2016; Safiye et al., 2023).

Given these challenges, there is a growing emphasis on supporting healthcare professionals in actively managing their mental health and overall well-being (Søvold et al., 2021). Self-care has been identified as an important protective factor, particularly in the context of high emotional demands and compassion fatigue (Kearney et al., 2009). Effective self-care involves monitoring one's emotional and stress levels and engaging in activities that support psychological recovery, such as relaxation techniques, mindfulness practices, physical activity, and social support (Pospos et al., 2018; Kearney et al., 2009). These strategies contribute to improved physical, mental, and emotional well-being and may enhance the ability of healthcare professionals to provide compassionate and effective care (Pospos et al., 2018; Kearney et al., 2009). In addition, psychological resilience and related constructs, such as the capacity for mentalizing, have been identified as important factors supporting adaptation to stressful and demanding work environments (Safiye et al., 2023).

Despite the recognized importance of self-care, healthcare professionals often face significant barriers to engaging in such activities. These barriers include time constraints, high workload, and concerns about stigma or perceived professional expectations (Safiye et al., 2023; Mills and Chapman, 2016). As a result, there is a need for accessible and flexible interventions that can support mental health in a practical and sustainable way.

Mobile health (mHealth) interventions have emerged as promising tools for delivering psychological support in an accessible and scalable manner (Firth et al., 2017; Weisel et al., 2019; Bakker et al., 2016). These interventions can help overcome barriers associated with traditional forms of support by providing flexible, self-guided, and cost-effective solutions. Previous research has reported positive outcomes associated with digital interventions, including reductions in burnout and improvements in mindfulness and psychological well-being (Monfries et al., 2023). However, despite rapid technological development, empirical evidence regarding their effectiveness among healthcare professionals remains limited.

Despite the growing body of research on digital mental health interventions, several important gaps remain. Existing evidence highlights considerable variability in intervention design, target populations, and methodological quality, limiting the ability to draw consistent conclusions about effectiveness (Cameron et al., 2025). Many studies rely on heterogeneous samples, which restricts the generalizability of findings to specific high-risk populations such as healthcare professionals.

In addition, although personalization has been identified as a promising strategy to enhance engagement and intervention outcomes, it remains insufficiently defined and inconsistently implemented in digital mental health interventions (Hornstein et al., 2023). Furthermore, relatively few studies simultaneously assess multiple key psychological outcomes—such as well-being, perceived stress, and burnout—using validated instruments within real-world healthcare settings, highlighting a critical gap in the current evidence base.

To address these limitations, the mobile application *CalmAnna* was developed as a personalized, evidence-informed digital intervention aimed at supporting mental well-being and reducing stress and burnout among healthcare professionals. The application integrates breathing exercises, mindfulness practices, relaxation techniques, and cognitive-behavioral strategies, and delivers tailored content based on user-reported inputs.

This study makes three key contributions to existing literature. First, it evaluates a personalized mHealth intervention tailored to individual psychological profiles, addressing the limited use of personalization in current digital interventions. Second, it simultaneously examines changes in well-being, perceived stress, and burnout using validated instruments within a single intervention framework. Third, it provides real-world evidence from healthcare professionals working in high-demand environments, a population that remains underrepresented in digital mental health research.

The present pilot study aimed to evaluate preliminary psychological outcomes associated with the use of the *CalmAnna* mobile application on mental well-being, perceived stress, and burnout among healthcare professionals. Based on prior evidence indicating that digital mindfulness and cognitive-behavioral interventions produce moderate to large effects in reducing stress and enhancing psychological well-being (e.g., Spijkerman et al., 2016; Heber et al., 2017; Burton et al., 2017), as well as findings suggesting that mHealth applications can be effective in healthcare populations (Alkhuzaimi et al., 2024; Moore et al., 2024), we hypothesized that, following six weeks of app use, participants would report increased mental well-being and decreased levels of perceived stress and burnout compared to baseline.

## 2 Methods

### 2.1 Study design

A single-group pre-post study design was employed to evaluate preliminary psychological outcomes associated with the use of the *CalmAnna* mobile application. Given the exploratory nature of this pilot study and the early stage of intervention development, a non-controlled design was considered appropriate to assess initial outcomes under real-world conditions. The intervention period lasted six weeks, with assessments conducted at baseline (T1) and immediately post-intervention (T2).

### 2.2 Participants

A convenience sample of healthcare professionals was recruited through a combination of institutional outreach and online dissemination. Healthcare facilities were contacted directly via email and invited to distribute study information among their staff. In addition, the study was promoted through professional networks and social media platforms targeting healthcare workers.

A total of 107 healthcare professionals were enrolled in the study, of whom 75 (70.1%) completed both baseline and post-intervention assessments and were included in the final analysis. Attrition analyses were conducted to compare participants who completed both assessments with those who did not complete the post-intervention assessment on available baseline demographic and psychological variables.

This multi-channel recruitment strategy was used to enhance accessibility and reach participants across different healthcare professions and settings.

Eligible participants were required to be at least 18 years of age, currently employed in a healthcare setting, own a smartphone (Android or iOS), and be willing to use the application for 6 weeks and complete both assessments. Individuals were excluded if they had a current severe psychiatric diagnosis or were participating in another digital or psychotherapeutic mental health program.

All participants provided written informed consent prior to enrollment in the study. The use of a convenience sampling approach reflects the exploratory nature of this pilot study and the aim to evaluate the intervention in a real-world healthcare context.

### 2.3 Intervention

The intervention was delivered using the *CalmAnna* self-guided mobile health (mHealth) application, a proprietary digital platform designed to support mental well-being and reduce stress and burnout among healthcare professionals. The intervention consisted of brief structured activities derived from evidence-based psychological approaches, including stress-management techniques, mindfulness-based practices, psychoeducational content, and cognitive-behavioral strategies. Individual activities were designed to be completed flexibly within daily routines and typically required approximately 5–15 minutes per session. Activities were organized into thematic content categories focusing on stress reduction, emotional regulation, cognitive coping strategies, and psychological recovery.

Upon initial use, participants completed an in-app self-assessment, which informed content selection within the application. This approach was intended to enhance user engagement and relevance of the intervention.

The application constitutes proprietary intellectual property currently subject to technology transfer and commercialization processes. Detailed technical, algorithmic, and design aspects are protected and are not publicly disclosed. At baseline, participants completed standardized assessments of stress, burnout, and well-being administered either online or via paper-and-pencil formats as part of the study protocol. In addition, an in-app self-assessment evaluating the same domains was used to generate a personalized intervention plan by prioritizing modules aligned with the user's psychological profile.

Participants were encouraged to use the application for approximately 10 min per day, 5–7 days per week, over a 6-week period. Push notifications were used to support adherence and remind users to engage with the intervention. No therapist guidance or external support was provided, and all activities were completed independently within the application.

Adherence to the intervention was not systematically assessed, as the mixed data collection approach (online and paper-based) did not allow for consistent linkage between questionnaire responses and app usage data at the individual level. Objective adherence metrics were available only in aggregated form and were not systematically analyzed at the individual participant level. Therefore, individual dose–response relationships between app usage and psychological outcomes could not be evaluated.

### 2.4 Measures

Participants completed a set of validated self-report instruments to assess mental well-being, perceived stress, burnout, and current psychological state. Internal consistency for each measure was evaluated using Cronbach's alpha based on the study sample.

**Mental well-being**. Mental well-being was assessed using the World Health Organization–Five Well-Being Index (WHO-5). The WHO-5 consists of five items reflecting positive mood, vitality, and general interest over the past 2 weeks. Items are rated on a 6-point Likert scale, with higher scores indicating greater well-being. The scale has demonstrated good psychometric properties across diverse populations. In the present study, internal consistency was high (Cronbach's α = 0.88) (Sischka et al., 2020).

**Perceived stress**. Perceived stress was measured using the 10-item Perceived Stress Scale (PSS-10; Cohen et al., 1983). The scale assesses the degree to which individuals perceive situations in their lives as stressful over the past month. Items are rated on a five-point Likert scale (zero = never to four = very often), with higher scores indicating higher levels of perceived stress. In this study, internal consistency was good (Cronbach's α = 0.87).

**Burnout**. Burnout was assessed using the Maslach Burnout Inventory (MBI; Maslach et al., 1996), a widely used instrument for evaluating occupational burnout. The MBI includes three subscales: emotional exhaustion, depersonalization, and personal accomplishment. Items are rated on a seven-point Likert scale (zer0 = never to six = every day). Higher scores on emotional exhaustion and depersonalization and lower scores on personal accomplishment indicate higher burnout. Internal consistency in the present study was high for emotional exhaustion (α = 0.93) and acceptable for depersonalization (α = 0.77) and personal accomplishment (α = 0.74).

**Psychological state**. Current psychological state was assessed using the SUPSO-7 (Mikšík, 2004), which captures both adaptive and maladaptive psychological states across seven domains, including depression, anxiety, activity, and well-being. Items are rated on a four-point scale. In this study, internal consistency across subscales was acceptable, with Cronbach's α ranging from 0.72 to 0.86.

**Sociodemographic variables**. Participants provided demographic and occupational information, including age, gender, level of education, profession, years of experience, work setting, and employment characteristics.

### 2.5 Procedure

Participants were enrolled in the study after providing written informed consent. At baseline (T1), they completed a set of standardized questionnaires assessing mental well-being (WHO-5), perceived stress (PSS-10), burnout (MBI), and current psychological state (SUPSO-7). Data collection was conducted using a combination of online and paper-and-pencil formats. Participants completing the online version accessed the questionnaires through the *CalmAnna* application, while paper-based questionnaires were distributed in participating healthcare facilities. This mixed-mode approach was used to increase accessibility across diverse work environments.

Following the baseline assessment, participants were instructed to use the *CalmAnna* mobile application over a 6-week intervention period. They were encouraged to engage with the application for approximately 10 min per day and to integrate its use into their daily routine. The intervention was self-guided and did not involve any therapist support.

At the end of the intervention period (T2), participants completed the same set of questionnaires to assess changes in mental well-being, perceived stress, burnout, and psychological state.

All procedures were conducted in accordance with the Declaration of Helsinki (World Medical Association, 2013). Participation was voluntary, and informed consent was obtained from all participants prior to data collection. No directly identifying personal information was collected. Participants were identified using self-generated unique codes, allowing pseudonymized matching of baseline and follow-up assessments without directly identifying participants. According to applicable national regulations and institutional guidance for non-interventional observational research using pseudonymized questionnaire data, formal ethics committee review or exemption was not required for the present pilot study. Participants were informed of their right to withdraw from the study at any time without any consequences.

### 2.6 Data processing and evaluation

Data were analyzed using Jamovi statistical software (version 2.6.44, The jamovi Project, Sydney, Australia). Descriptive statistics were calculated for all continuous variables and are presented as means and standard deviations, while categorical variables are reported as absolute and relative frequencies. Only participants who completed both baseline and post-intervention assessments were included in the final analyses. No imputation procedures were applied. Attrition analyses were conducted to compare participants who completed both assessments with those who did not complete the post-intervention assessment on available baseline demographic and psychological variables.

Pre–post changes in psychological outcomes were examined using repeated-measures analysis of variance (RM-ANOVA). Given that only two time points were assessed (baseline and post-intervention), RM-ANOVA is mathematically equivalent to a paired-samples *t*-test. Therefore, paired *t*-tests were additionally reported to provide estimates of mean differences, 95% confidence intervals (CIs), and Cohen's *d* effect sizes to facilitate interpretation of the magnitude of change.

Effect sizes for repeated-measures ANOVA were expressed as partial eta squared (η*p*^2^), while effect sizes for paired-sample comparisons were calculated using Cohen's *d* for dependent samples (*dz*). Cohen's *d* values were interpreted according to conventional benchmarks (small = 0.2, medium = 0.5, large = 0.8).

The assumption of normality was assessed using the Shapiro–Wilk test. Although several variables showed significant deviations from normality, parametric tests were retained due to the sample size (*n* = 75) and the robustness of parametric methods to violations of normality under such conditions. In accordance with the central limit theorem, the sampling distribution of the mean approaches' normality with increasing sample size, allowing reliable use of parametric tests even when raw data are not normally distributed. Sensitivity analyses using Wilcoxon signed-rank tests were conducted for variables violating the normality assumption in order to assess the robustness of the findings.

To control for multiple comparisons across secondary outcomes (PSS, MBI subscales, and SUPSO dimensions), the false discovery rate (FDR) correction using the Benjamini–Hochberg procedure was applied. The primary outcome (WHO-5) was not adjusted for multiple testing.

All statistical tests were two-tailed, and the level of statistical significance was set at α = 0.05.

## 3 Results

### 3.1 Participant characteristics

A total of 75 healthcare professionals were included in the final analysis. The sample comprised predominantly women (88%), with a mean age of 37.56 years (SD = 13.55). Participants had an average of 13.95 years of professional experience (SD = 13.69) and had been working at their current workplace for 8.76 years on average (SD = 9.33).

Most participants were non-physician healthcare workers (76%), while 24% were physicians. Most participants worked in the public sector (93.3%), and approximately one-quarter held a leadership position (22.7%).

Further details are presented in Table 1. To evaluate potential attrition bias, baseline characteristics of participants who completed both assessments were compared with those of non-completers; the results are summarized in Table 2.

**Table 1:** Characteristic of research sample.

Characteristic of research sample	No. (%) (*n* = 75)
Sex – no. (%)	
Male	9 (12.0)
Female	66 (88.0)
Education level – no. (%)	
Secondary education w/exam	35 (46.7)
Higher specialized education	3 (4.0)
College undergraduate degree – BS, BA.	11 (14.7)
College graduate degree	26 (34.7)
Job position – no. (%)	
Physician	18 (24.0)
Non-medical healthcare worker	57 (76.0)
Sector – no. (%)	
State	70 (93.3)
Private	5 (6.7)
Work schedule– no. (%)	
A dayshift	45 (60.0)
two-shift regimen	25 (33.3)
three-shift regimen	5 (6.7)
Lead worker– no. (%)	
Yes	17 (22.7)
No	58 (77.3)
Characteristic	Mean ± SD
Age – year	37.56 ± 13.55
Length of employment – year	13.95 ± 13.69
Length of employment at current workplace – year	8.76 ± 9.33

**Table 2:** Baseline comparison of completers and non-completers.

Variable	Completers (*n* = 75)	Non-completers (*n* = 32)	*t*/χ^2^	*p*	Cohen's *d*
Age	37.56 (13.55)	36.16 (14.20)	0.48	0.630	0.10
Female, *n* (%)	66 (88.0)	27 (84.4)	0.26	0.611	—
WHO-5 baseline	11.60 (4.20)	13.63 (4.91)	−2.17	0.032	−0.46
SUPSO-S (psychological distress)	5.47 (2.71)	3.84 (2.05)	3.20	0.002	0.68
Perceived stress (PSS)	21.49 (7.56)	20.28 (8.97)	0.72	0.475	0.15
Emotional exhaustion (MBI-EE)	29.57 (15.17)	31.84 (14.38)	−0.72	0.473	−0.15

Continuous variables are presented as means with standard deviations in parentheses. Group differences for continuous variables were examined using independent-samples *t* tests; gender differences were examined using the chi-square test. WHO-5, World Health Organization–Five Well-Being Index; SUPSO-S, psychological distress dimension of the SUPSO; PSS, Perceived Stress Scale; MBI-EE, Maslach Burnout Inventory–Emotional Exhaustion subscale.

Attrition analyses comparing completers and non-completers revealed significant baseline differences in well-being, *t*(105) = −2.17, *p* = 0.032, *d* = −0.46, and in the SUPSO-S dimension reflecting psychological distress and discomfort, *t*(105) = 3.20, *p* = 0.002, *d* = 0.68. Non-completers reported higher baseline well-being and lower psychological distress. No significant differences were observed in age, gender distribution, perceived stress, or burnout indicators.

### 3.2 Changes in psychological outcomes

Mental well-being increased from baseline (*M* = 11.60, SD = 4.20) to post-intervention (*M* = 13.15, SD = 5.48), with a mean change of 1.55 points (95% CI [0.61, 2.48]), indicating a reliable improvement. This change was statistically significant, *F*(1,74) = 10.90, *p* = 0.001, η*p*^2^ = 0.128.

Perceived stress decreased from 21.49 (SD = 7.56) to 19.76 (SD = 7.01), with a mean reduction of 1.73 points (95% CI [−2.40, −1.06]), indicating a consistent decrease across participants. This change was statistically significant, *t*(74) = −5.16, *p* < 0.001.

Burnout indicators showed consistent improvements. Emotional exhaustion decreased by 2.11 points (95% CI [−2.96, −1.25]), and depersonalization decreased by 1.13 points (95% CI [−1.55, −0.72]). Personal accomplishment increased by 0.83 points (95% CI [0.10, 1.56]). All changes were statistically significant.

Across SUPSO dimensions, both positive and negative psychological states showed consistent changes. Positive dimensions increased (SUPSO-P: +0.56, 95% CI [0.27, 0.85]; SUPSO-A: +0.71, 95% CI [0.47, 0.94]), while negative dimensions decreased (e.g., SUPSO-S: −1.17, 95% CI [−1.70, −0.65]; SUPSO-D: −0.88, 95% CI [−1.23, −0.53]). These confidence intervals did not include zero, indicating consistent changes across participants.

To control for multiple comparisons, false discovery rate (FDR) correction was applied to secondary outcomes, with all results remaining statistically significant.

Pre–post descriptive statistics are presented in Table 3, and inferential analyses are summarized in Table 4. Sensitivity analyses using Wilcoxon signed-rank tests yielded results consistent with the primary parametric analyses, with all statistically significant findings remaining significant under non-parametric testing.

**Table 3:** Pre-post descriptive statistics (*n* = 75).

Outcome	Pre Mean ±SD	Post Mean ±SD	Mean change (Post–Pre)	95% CI low	95% CI high	Direction
**WHO-5**	11.600 ± 4.195	13.147 ± 5.484	1.547	0.613	2.480	Improvement
**PSS**	21.493 ± 7.561	19.760 ± 7.009	−1.733	−2.403	−1.064	Improvement
MBI						
EE	29.573 ± 15.168	27.467 ± 14.847	−2.107	−2.963	−1.250	Improvement
DP	11.253 ± 6.498	10.120 ± 6.499	−1.133	−1.550	−0.717	Improvement
PA	30.453 ± 8.214	31.280 ± 8.627	0.827	0.097	1.557	Improvement
SUPSO						
P-Mental well-being	6.347 ± 2.469	6.907 ± 2.717	0.560	0.271	0.849	Improvement
A-Active	5.573 ± 2.445	6.280 ± 2.414	0.707	0.471	0.942	Improvement
*O*-Impulsivity	4.493 ± 2.528	3.760 ± 2.364	−0.733	−1.015	−0.452	Improvement
*N*-Mental restless	5.587 ± 2.742	4.947 ± 2.726	−0.640	−0.996	−0.284	Improvement
*U*-Anxiety	7.187 ± 3.092	6.493 ± 3.663	−0.693	−1.058	−0.329	Improvement
*D*-Depression	5.947 ± 3.110	5.067 ± 3.112	−0.880	−1.229	−0.531	Improvement
*S*-Sadness	6.467 ± 2.606	5.293 ± 3.384	−1.173	−1.701	−0.646	Improvement

**Table 4:** Main inferential analyses (*n* = 75).

Outcome	RM-ANOVA F(1,74)	*ηp* ^2^	Paired *t*	*p*	FDR *p* (secondary)	Cohen *dz*	Direction
**WHO-5**	10.895	0.128	3.301	0.001		0.381	Improvement
**PSS**	26.609	0.264	−5.158	< 0.001	< 0.001	−0.596	Improvement
MBI							
EE	24.027	0.245	−4.902	< 0.001	< 0.001	−0.566	Improvement
DP	29.376	0.284	−5.420	< 0.001	< 0.001	−0.626	Improvement
PA	5.093	0.064	2.257	0.027	0.027	0.261	Improvement
SUPSO							
P-Mental well-being	14.942	0.168	3.866	< 0.001	< 0.001	0.446	Improvement
A-Active	35.740	0.326	5.978	< 0.001	< 0.001	0.690	Improvement
O-Impulsivity	26.970	0.267	−5.193	< 0.001	< 0.001	−0.600	Improvement
N-Mental restless	12.823	0.148	−3.581	< 0.001	< 0.001	−0.413	Improvement
U-Anxiety	14.348	0.162	−3.788	< 0.001	< 0.001	−0.437	Improvement
D-Depression	25.294	0.255	−5.029	< 0.001	< 0.001	−0.581	Improvement
S-Sadness	19.655	0.210	−4.433	< 0.001	< 0.001	−0.512	Improvement

η*p*^2^, partial eta squared.

## 4 Discussion

This pilot study found that regular use of *CalmAnna* was associated with significant improvements in well-being and reductions in stress and burnout among healthcare professionals. These findings are consistent with the study hypothesis, as participants reported improvements in mental well-being alongside reductions in perceived stress and burnout following the intervention period. The findings indicate that even a brief, self-guided digital intervention may be associated with measurable psychological benefits in a population that is chronically exposed to emotional strain and occupational overload. The results are consistent with previous digital and mHealth studies demonstrating the benefits of mindfulness- and CBT-based interventions (Spijkerman et al., 2016; Heber et al., 2017; Burton et al., 2017) and extend this evidence by showing that improvements occurred across a broad range of psychological indicators.

Mental well-being increased reliably, while perceived stress and all core dimensions of burnout—emotional exhaustion, depersonalization, and personal accomplishment—showed significant and consistent changes. These patterns suggest that *CalmAnna* may influence both negative and positive psychological processes. The observed improvements in SUPSO dimensions further support this interpretation: positive states increased, whereas negative states decreased, with all confidence intervals excluding zero. This uniform direction of change indicates a global shift in psychological functioning rather than isolated improvements in specific domains. When converted to the standard 0–100 WHO-5 scale, the observed improvement corresponded to approximately six points. Although statistically significant, this change remained below the commonly referenced threshold for clinically meaningful improvement, suggesting that the findings should be interpreted as preliminary and primarily indicative of early change rather than established clinical effectiveness.

The application includes adaptive content features informed by initial user input, which may have contributed to enhanced engagement and perceived relevance of the intervention (Kearney et al., 2009; Hornstein et al., 2023). The observed effect sizes ranged from small to moderate across outcomes and were broadly consistent with prior digital interventions targeting psychological well-being (Mills and Chapman, 2016; Firth et al., 2017), reinforcing the potential of brief digital tools to support mental health in demanding clinical environments.

The mechanisms underlying the observed effects were not directly assessed in the present study and therefore remain an important area for future research. Nevertheless, several general processes may help explain the observed improvements. Digital interventions drawing on evidence-based psychological principles have been shown to reduce cognitive load, enhance emotional regulation, and interrupt maladaptive stress responses. Similar mechanisms have been reported in previous research: mindfulness-based programs for healthcare workers have been associated with improved emotional regulation and reduced cognitive overload (e.g., Krasner et al., 2009; West et al., 2018), while digital cognitive-behavioral interventions have demonstrated effectiveness in disrupting maladaptive stress patterns and reducing burnout symptoms (Heber et al., 2017; Stratton et al., 2017). The consistent reduction in burnout indicators observed in the present study aligns with these findings and suggests that the intervention may help mitigate chronic emotional exhaustion and cynicism—two core drivers of healthcare worker distress. Evidence from other mHealth studies further supports this interpretation, indicating that brief, app-based interventions can produce meaningful improvements in both negative and positive psychological states among clinical staff (Burton et al., 2017; Lomas et al., 2018).

Strengths of the study include the use of validated instruments, internal reliability assessment (Cronbach's α ≥ 0.74 across scales), and the assessment of multiple psychological outcomes relevant to healthcare professionals.

The study also has several limitations that should be considered. Given the single-group pre–post design, the observed changes cannot be interpreted as causal effects of the intervention, as alternative explanations such as regression to the mean, expectancy effects, or natural fluctuations in psychological states cannot be ruled out.

The small, self-selected sample limits the generalizability of the findings, and the absence of a control group precludes causal inference. Participants may have been more motivated or interested in mental health interventions than the general population of healthcare professionals, which may limit the external validity of the findings. In addition, attrition analyses suggested that participants with higher baseline well-being were less likely to complete the post-intervention assessment, which may have influenced the observed magnitude of change.

The short follow up period does not allow conclusions regarding the long-term sustainability of the effects, and reliance on self-report measures introduces potential bias. A key limitation of the study is the absence of objective adherence data, which prevents precise evaluation of the dose–response relationship between app usage and observed outcomes. Future randomized controlled trials should therefore include larger and more diverse samples, objective usage data, and extended follow up periods to assess the durability of outcomes. It would also be valuable to examine mediators of change—such as emotion regulation, cognitive reappraisal, or perceived control—to gain a clearer understanding of how digital interventions exert their effects. However, these proposed mechanisms remain speculative and were not directly assessed in the present study. In addition, the study was not preregistered, which should be considered a limitation of the exploratory pilot design.

Despite these limitations, the overall pattern of results indicates that *CalmAnna* demonstrates potential as a scalable, low-cost preventive tool for supporting the mental health of healthcare workers and complementing institutional wellness initiatives. Importantly, the study provides ecologically valid evidence from a real-world healthcare setting, which is often underrepresented in controlled intervention research. Future research should incorporate randomized controlled designs, objective usage metrics, and longer follow-up periods to better evaluate the effectiveness and sustainability of digital mental health interventions.

## 5 Conclusions

*CalmAnna* appears to be a promising mHealth intervention associated with improvements in well-being and reductions in stress and burnout among healthcare professionals. Psychometric analyses confirmed reliable outcome measurement, and the observed pattern of results supports further controlled evaluation.

While further controlled research is needed, *CalmAnna* represents a promising digital approach for supporting psychological well-being in the healthcare workforce. Taken together, the findings suggest that such interventions may have the potential to serve as scalable, low-cost tools that complement institutional mental health strategies.

## Data Availability

Data are shared in ZENODO repository doi: https://doi.org/10.5281/zenodo.20716957.
